# Using multi-way admixture mapping to elucidate TB susceptibility in the South African Coloured population

**DOI:** 10.1186/1471-2164-15-1021

**Published:** 2014-11-25

**Authors:** Michelle Daya, Lize van der Merwe, Christopher R Gignoux, Paul D van Helden, Marlo Möller, Eileen G Hoal

**Affiliations:** Molecular Biology and Human Genetics, MRC Centre for TB Research and the DST/NRF Centre of Excellence for Biomedical TB Research, Faculty of Medicine and Health Sciences, Stellenbosch University, Tygerberg, South Africa; Department of Genetics, Stanford University, Stanford, California USA

**Keywords:** Local ancestry inference, 15q15, 17q22, GADD45A, OSM, B7-H5

## Abstract

**Background:**

The admixed South African Coloured population is ideally suited to the discovery of tuberculosis susceptibility genetic variants and their probable ethnic origins, but previous attempts at finding such variants using genome-wide admixture mapping were hampered by the inaccuracy of local ancestry inference. In this study, we infer local ancestry using the novel algorithm implemented in RFMix, with the emphasis on identifying regions of excess San or Bantu ancestry, which we hypothesize may harbour TB susceptibility genes.

**Results:**

Using simulated data, we demonstrate reasonable accuracy of local ancestry inference by RFMix, with a tendency towards miss-calling San ancestry as Bantu. Regions with either excess San ancestry or excess African (San or Bantu) ancestry are less likely to be affected by this bias, and we therefore proceeded to identify such regions, found in cases but not in controls (642 cases and 91 controls). A number of promising regions were found (overall p-values of 7.19×10^-5^ for San ancestry and <2.00×10^-16^ for African ancestry), including chromosomes 15q15 and 17q22, which are close to genomic regions previously implicated in TB. Promising immune-related susceptibility genes such as the *GADD45A*, *OSM* and *B7-H5* genes are also harboured in the identified regions.

**Conclusion:**

Admixture mapping is feasible in the South African Coloured population and a number of novel TB susceptibility genomic regions were uncovered.

**Electronic supplementary material:**

The online version of this article (doi:10.1186/1471-2164-15-1021) contains supplementary material, which is available to authorized users.

## Background

The South African Coloured population (SAC) is a so-called admixed population that derived its origins from the diverse population groups that settled in the early Cape colony, including the indigenous San, early European settlers, slaves that were imported from Indonesia, India and other parts of Africa, and South African Bantu-speakers who later migrated to the area. Previous genetic research has shown that the SAC received ancestry contributions from click-speaking Africans (San), Bantu-speaking Africans, Europeans and South and East Asians, which is consistent with the historical records [1–5]. A high degree of heterogeneity in ancestral contributions between SAC individuals has also been illustrated previously [1, 2, 6]. The admixture that occurred in the SAC is therefore complex, constituting a number of different source ancestries, with dissimilar genetic distances between them.

Our study group of SAC individuals was recruited from metropolitan areas in Cape Town that have some of the highest reported incidences of tuberculosis (TB) worldwide, despite extensive BCG vaccination and low prevalence of HIV [7]. As the group received contributions from diverse source populations that may differ in their genetic susceptibility to TB, the group is ideally suited to the discovery of TB susceptibility genetic variants and their probable ethnic origins. Our previous work has shown that African ancestry in this group is associated with higher risk of TB infection, whereas European and Asian ancestries are protective [8, 9]. Areas of the genome with African ancestry that is much higher than the norm in a group of TB cases may therefore harbour genetic variants that increase the risk of developing TB. The process of finding such areas is known as admixture mapping, and this technique relies on the accurate inference of what is known as local ancestry per individual across their genome [10].

When admixture occurs between two or more population groups that were previously isolated, recombination events result in chromosomes that are a mosaic of blocks of ancestry deriving from different source populations. Given genetic data of an admixed individual and their source populations, statistical techniques can be used to determine the bounds of these segments and to assign the most probable source ancestries to them. These techniques rely on the probability of recombination events to distinguish the bounds of segments, and differences in allele and haplotype frequencies between source populations for classification of the ancestry of segments. The process is known as local ancestry inference (LAI).

In a previous study, Chimusa et al. concluded that accurate multi-way LAI was not feasible in the SAC using the LAI algorithms available at the time [9]. In this study, we re-evaluate this position using the novel LAI algorithm implemented in the RFMix software package, focusing on the classification of San and Bantu ancestry. These ancestries are of particular interest as Southern African populations were not exposed to modern strains of *Mycobacterium tuberculosis* (*M. tuberculosis*), the most prevalent in our SAC study group [11], until the recent past [12]. The relative lack of exposure of the SAC and Bantu populations to modern strains of *M. tuberculosis* could possibly have resulted in decreased resistance to developing the disease, especially in densely populated areas with low socio-economic conditions. Supporting this argument, a significant positive association between San ancestry and TB susceptibility in the SAC was found by Chimusa et al. [9]. The association was confirmed in an independent sample in a later study, which also found a positive association with Bantu ancestry, although it was relatively weak [8]. Although each of the non-African ancestry components of the SAC (European, South Asian and East Asian) were negatively associated with TB susceptibility when tested in individual models, these associations were no longer significant when all five ancestry components were tested together.

In this study, we first explore the accuracy of LAI in the SAC using RFMix and compare its performance to other algorithms. After quantifying this using simulated data, we proceed to identify regions with excess San or Bantu ancestry found in TB cases but not in controls, and hypothesize that these regions may contain genes that affect TB susceptibility.

## Results

### Evaluating multi-way LAI accuracy using simulated data

Chimusa et al. previously evaluated the accuracy of LAI in the SAC using various software programs and found that LAMP-LD performed best [9]. As RFMix was not available at that time, our first step was to compare the accuracies of LAMP-LD and RFMix using a simulated data set of 1500 SAC chromosomes (chromosome 1). LAI was run five-way, but since only San and Bantu genome-wide ancestry is independently associated with TB susceptibility [8, 9], ancestry of SNPs that were called as European, South Asian or East Asian were labelled as non-African. The percentage of SNPs for which the called ancestry matched the known ancestry was 69.43% and 96.43% for LAMP-LD and RFMix respectively. The percentage of SNPs per type of miss-called ancestry is summarized in Table 1, which shows that RFMix offers a significant improvement in the calling of local ancestry, especially when distinguishing San and Bantu ancestry.

**Table 1:** **Percentage of miss-called ancestry**

	Percentage of total [IQR]		Percentage of Ancestry [IQR]	
Type of miss-call	LAMP-LD	RFMix	LAMP-LD	RFMix
San as Bantu	4.10 [1.65–9.76]	1.95 [0.88–3.55]	13.28 [5.12–35.16]	6.19 [3.13–10.45]
San as non-African	1.11 [0.05–9.58]	0.27 [0.11–0.66]	3.12 [0.18–36.09]	0.89 [0.36–2.05]
Bantu as San	0.08 [0.00–5.49]	0.04 [0.00–0.13]	0.32 [0.00–21.54]	0.14 [0.00–0.49]
Bantu as non-African	0.45 [0.02–8.63]	0.09 [0.02–0.23]	2.36 [0.07–33.44]	0.37 [0.09–0.92]
Non-African as San	0.14 [0.00–8.31]	0.09 [0.03–0.19]	0.42 [0.00–25.87]	0.25 [0.08–0.54]
Non-African as Bantu	0.95 [0.14–9.66]	0.18 [0.07–0.33]	3.09 [0.35–28.68]	0.47 [0.20–0.92]

This table reports the interquartile range (IQR) of the percentage of SNPs that were miss-called by LAMP-LD and RFMix per each of the six possible miss-call categories. The known ancestry of a simulated data set of 1500 SAC chromosomes was compared to the ancestry called by the software program (chromosome 1). The median percentage of miss-called SNPs across all SNPs as well as the median percentage of miss-called SNPs across SNPs of that source ancestry are shown. San ancestry can for example be miss-called as either Bantu or non-African ancestry. The median percentage of all SNPs that were miss-called as such are shown in the second and third columns of the first two rows, and the median percentage of San SNPs that were miss-called as such are shown in the fourth and fifth columns of the first two rows. The mean proportion of San, Bantu and non-African ancestry in the simulated data set was 0.3342, 0.2772 and 0.3885 respectively. The difference in number of SNPs miss-called by RFMix, compared to the corresponding number of SNPs miss-called by LAMP-LD, were significant with p-values ***<***2***×***10^***-***16^ for each of the six possible miss-call categories.

Histograms of the difference between the mean ancestry called by RFMix per chromosome and the known mean ancestry in the simulated data set are shown in Additional file 1: Figure S1. Ancestry called by RFMix was on average 2.71% lower than the known San ancestry, whilst Bantu ancestry was on average 2.45% higher. This discrepancy can be ascribed to the relatively large proportion of San SNPs that were miss-called as Bantu. Non-African ancestry calls compared well to known values (on average only 0.26% higher).

Could chromosomal segments with large deviations from the mean ancestry be the result of LAI errors? To answer this question, the simulated chromosome 1 data set was divided into segments by determining the positions where ancestry switches occur (see *Methods* - *Delineating called ancestry segments*), yielding 1077 segments. Deviation from the overall RFMix mean ancestry was calculated for each of the ancestries, for each of the segments. Table 2 summarizes the correlation between the number of miss-called ancestry segments and deviation in ancestry. Segments with lack of San ancestry are associated with San segments that are miss-called as Bantu or non-African, whereas segments with excess Bantu ancestry are associated with Bantu segments miss-called as San. The relationship between number of errors in segments and deviation in ancestry are further depicted in Additional file 1: Figures S2, S3 and S4. Although errors appear to be distributed fairly evenly across the entire chromosome, segments with lack of San ancestry generally have more errors. The exception is a large number of errors that occurred around the centromere (Additional file 1: Figure S4), likely due to the dearth of SNPs in this region.

**Table 2:** **Correlation between the number of miss-called ancestry segments and deviation in ancestry**

	Deviation		
Number miss-called	San	Bantu	Non-African
San as Bantu	-0.83	+0.89	+0.05
San as non-African	-0.39	+0.19	+0.43
Bantu as San	+0.01	+0.09	-0.17
Bantu as non-African	-0.02	+0.13	-0.21
Non-African as San	+0.01	+0.06	-0.14
Non-African as Bantu	-0.11	+0.24	-0.21

This table summarizes the correlation between the number of ancestry miss-calls that occurred at a segment of ancestry, per each of the six possible miss-call categories, and the deviation in local ancestry of the segment. Miss-called ancestry was identified by comparing the known ancestry of a simulated data set of 1500 SAC chromosomes to the ancestry called by RFMix (chromosome 1). Deviations in ancestry were calculated by subtracting the overall mean RFMix ancestry from the local ancestry of each segment, for each of the three source ancestries (San, Bantu, non-African).

The negative effect that short tracts of ancestry and a large degree of admixture could have on LAI accuracy was explored next. Additional file 1: Figure S5 shows the distribution of the length of tracts of ancestry in the simulated data and the proportion of SNPs with miss-called ancestry per tract. Tracts that were completely miss-called (all the SNPs in the tract were assigned incorrect ancestry by the LAI) occurred far more frequently in very short tracts of ancestry, and longer tracts of ancestry correlated with a smaller proportion of miss-called SNPs (r = -0.2906, p-value <2.00×10^-16^). Additional file 1: Figure S6 shows that there is a positive correlation between the number of tracts of ancestry on a chromosome and the number of miss-called SNPs for that chromosome (r = 0.1847, p-value =2.54×10^-13^), indicating that inferring local ancestry may be more error-prone for chromosomes with a large degree of admixture.

### Local ancestry across the genome

**Figure 1:**
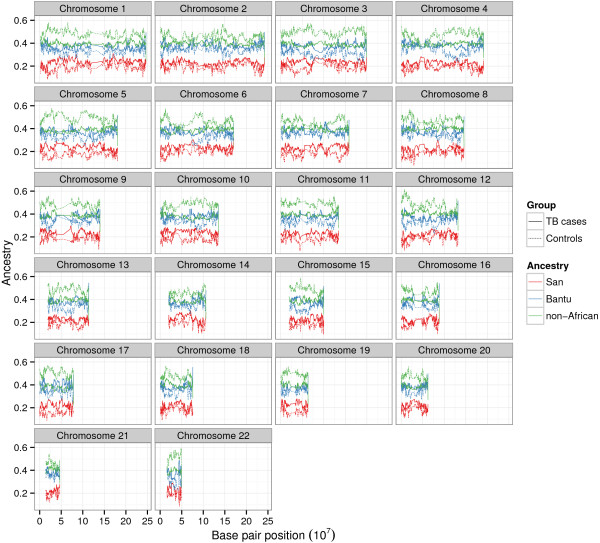
**Mean local ancestry across the genome.** The mean local ancestry estimates of TB cases and controls are shown per genomic position, for each of the source ancestries. Each panel represents a separate chromosome.

We proceeded to run LAI on our study group of 733 unrelated SAC individuals using RFMix (642 TB cases and 91 controls). Figure 1 depicts local ancestry across the genome for cases and controls. The mean genome-wide San ancestry calculated from the local ancestry estimates was 0.2304 and 0.1847 for cases and controls respectively, the mean Bantu ancestry was 0.3792 (cases) and 0.3391 (controls), and the mean non-African ancestry was 0.3904 (cases) and 0.4761 (controls). Mean San ancestry was on average 12.10% lower than corresponding ADMIXTURE estimates and Bantu ancestry was on average 10.80% higher, whilst the mean non-African ancestry was comparable (only 1.31% higher). The differences in ancestry estimates are illustrated in Additional file 1: Figure S7. We speculate that the large discrepancy between San and Bantu estimates can in part be ascribed to some of the San ancestry in the SAC being contributed by southern African Bantu populations such as the Xhosa [1, 2]. Admixture between the San and these populations likely occured during the Bantu expansion [13]. The relative older age of these admixture events would result in short tracts of San ancestry, which are harder to distinguish with genotype array data [14], also evident from our simulated data (Additional file 1: Figure S5). The distributions of called San and European tract lengths are comparable, supporting our conclusion that very short tracts of San ancestry may not have been identified, whereas the Bantu tract lengths are longer (Additional file 1: Figure S8). Xhosa admixture into the nascent SAC population likely occurred later than admixture between the San and Europeans [1], helping to explain the longer Bantu tract lengths.

RFMix output was divided into 13 860 segments of local ancestry and local ancestry deviations were calculated. Histograms of the local ancestry deviations in cases and controls are depicted in Additional file 1: Figure S9 and boxplots of the deviations are shown in Additional file 1: Figure S10. Similar to our findings in simulated data, the figures suggest that the tails of the deviation distributions are biased towards lack of San ancestry and excess Bantu and non-African ancestry.

### Regions with excess San or Bantu ancestry in cases relative to controls

Previous work has shown that San and Bantu ancestry is associated with increased susceptibility to TB in the SAC, and that the non-African ancestry components (European, South Asian and East Asian) are protective. The non-African ancestry components were however not associated with TB susceptibility after adjustment for the other ancestry components, whereas the San and Bantu components remained significant. The positive association between San ancestry and TB susceptibility was highly significant, whereas association with Bantu ancestry was relatively weak (p-value =1.06×10^-11^ and p-value =3.00×10^-2^ respectively) [8]. We were therefore interested in finding regions of the genome with excess San or Bantu ancestry in cases, but not in controls. From our simulations and analysis of the distribution of local ancestry deviations in our study group, it is evident that excess Bantu ancestry may be enriched with miss-called ancestry. As this is not the case for excess San ancestry, or excess San or Bantu ancestry (i.e. lack of non-African ancestry), we used two joint models to test for differences in ancestry between cases and controls per ancestry segment. One model tested for difference in San ancestry, and the other tested for differences in African (San or Bantu) ancestry. Only segments with ancestry two standard deviations above the genome-wide mean in cases were included in the models (110 San segments and 238 African segments), and age, gender and genome-wide ancestry were adjusted for, yielding p-values of 7.19×10^-5^ (San) and <2.00×10^-16^ (Bantu). Regions that differ significantly between cases and controls are summarized in Tables 3 and 4. The regions are comprised of a number of contiguous ancestry segments, and individual p-values for these sub-regions are summarized in Additional file 1: Tables S1 and S2, ranging between 0.0465 and 0.0005. Some of the segments in the African analysis also have large differences in San ancestry (e.g. some of the chromosome 10 and 17 segments have 7% or more San ancestry in TB cases compared to controls, which is comparable to the differences detected in the San ancestry model). These segments were however not detected in the San analysis as the mean proportion of San ancestry in TB cases fell just short of the mean plus two standard deviation cut-off for inclusion in the San ancestry model (mean + 2SD = 0.2820). On chromosome 17, 10 segments with the smallest p-values gave estimated TB case-control odds ratios of having African ancestry versus any other ancestry ranging from 1.77 (95% CI:1.27–2.46) to 1.61 (95% CI: 1.16–2.24). (The odds ratio for each segment is the estimated odds of having TB versus not having TB for an African segment, compared to the odds for a non-African segment.)

**Table 3:** **Regions of the genome with excess San ancestry in TB cases relative to controls**

Length			Mean San ancestry		
Region	Begin-end SNP	(Nr SNPs)	TB cases	Control	Genes
1p31	rs12144711-rs7554551	671230 (123)	0.2902	0.1615	GADD45A, GNG12, DIRAS32
9q21	rs2309428-rs1847503	2080640 (323)	0.2909	0.1609	FAM189A, APBA1, PTAR1, C9orf135, MAMDC2, SMC5, KLF9
22q12	rs16986925-rs6006426	1290997 (152)	0.2850	0.1745	C22orf31, KREMEN1, EWSR1, RHBDD3, EMID1, AP1B1, RASL10A, GAS2L1, NEFH, RFPL1, NF2, NIPSNAP1, THOC5, UQCR10, CABP7, ZMAT5, ASCC2, MTMR3, HORMAD2, LIF, OSM

This table summarizes regions of the genome with excess San ancestry, found in TB cases relative to controls, after adjusting for age, gender and genome-wide San ancestry. Ancestry segments that are associated with increased San ancestry in cases compared to controls were identified and contiguous segments were merged. P-values for each of the individual ancestry segments are available in Additional file 1: Table S1. The mean RFMix genome-wide San ancestry estimates are 0.2304 and 0.1847 for cases and controls respectively, and the standard deviation of San local ancestry deviations is 0.0258 and 0.0321 in cases and controls respectively. Only regions of 500 000 base pairs or longer are shown (two short regions on chromosome 5 were excluded).

**Table 4:** **Regions of the genome with excess African ancestry in TB cases relative to controls**

Length			Mean African ancestry		
Region	Begin-end SNP	(Nr SNPs)	TB cases	Control	Genes
5q11	rs26090-rs1382907	739064 (70)	0.6480	0.4615	ISL1
10q22	rs827299-rs7083934	6243529 (693)	0.6607	0.5030	UNC5B, SLC29A3, CDH23, C10orf105, PSAP, B7-H5, CHST3, SPOCK2, ASCC1, DDIT4, DNAJB12, MICU1, MCU, OIT3, PLA2G12B, P4HA1, NUDT13, FAM149B1, DNAJC9, MRPS16, TTC18, ANXA7, PPP3CB, MSS51, MYOZ1, AGAP5, SYNPO2L, CAMK2G, NDST2, SEC24C, ZSWIM8, FUT11, CHCHD1, PLAU, C10orf55, VCL, AP3M1, ADK, KAT6B, DUPD1, SAMD8, DUSP13, VDAC2, COMTD1, ZNF503
15q15	rs1712435-rs16966424	2669916 (182)	0.6511	0.4963	PLA2G4D, VPS39, GANC, TMEM87A, CAPN3, SNAP23, ZNF106, HAUS2, LRRC57, TTBK2, CDAN1, UBR1, EPB42, TMEM62, TGM5, TGM7, TP53BP1, LCMT2, ZSCAN29, TUBGCP4, ADAL, CKMT1B, MAP1A, PPIP5K1, STRC, CKMT1A, CATSPER2, PDIA3, MFAP1, SERF2, HYPK, ELL3, SERINC4, WDR76, FRMD5, CASC4, CTDSPL2, EIF3J, SPG11, PATL2
17q22	rs7210845-rs9908090	5200677 (479)	0.6579	0.4698	ANKFN1, NOG, C17orf67, TRIM25, DGKE, COIL, SCPEP1, AKAP1, MSI2, CCDC182, MRPS23, CUEDC1, SRSF1, VEZF1, DYNLL2, EPX, OR4D1, MKS1, OR4D2, LPO, MPO, BZRAP1, SUPT4H1, RNF43, HSF5, SEPT4, MTMR4, C17orf47, TEX14, RAD51C, PPM1E, TRIM37, SKA2, GDPD1, SMG8, PRR11, CLTC, DHX40, PTRH2, VMP1, RPS6KB1, TUBD1, RNFT1, HEATR6, CA4, USP32, SCARNA20, RPL32P32, C17orf64, APPBP2, PPM1D

This table summarizes regions of the genome with excess African ancestry (San or Bantu), found in TB cases relative to controls, after adjusting for age, gender and genome-wide African ancestry. Ancestry segments that are associated with increased African ancestry in cases compared to controls were identified and contiguous segments were merged. P-values for each of the individual ancestry segments are available in Additional file 1: Table S2. The mean RFMix genome-wide African ancestry estimates are 0.6096 and 0.5238 for cases and controls respectively, and the standard deviation of local ancestry deviations is 0.0187 and 0.0336 in cases and controls respectively. Only regions of 500 000 base pairs or longer and that contain protein coding genes are shown (one short region on chromosome 5, one short region on chromosome 6, and four short regions on chromosome 10 were excluded).

## Discussion

Previously it was thought that admixture mapping in the SAC was not feasible using available LAI methods, due to the complex five-way admixture that occurred in this population [9]. Using the novel LAI algorithm implemented in RFMix, and by focusing on finding regions with excess San or excess San or Bantu ancestry in TB cases relative to controls, we have demonstrated that this technique can be applied to our SAC TB susceptibility study group. By limiting our tests for associations to only these directional ancestry components, and by using only two models to test for excess ancestry in TB cases relative to controls, we also reduced our study’s vulnerability to false positive association signals that can arise as a result of multiple testing.

Based on their putative role in TB pathogenesis, we identified a number of genes in regions of excess ancestry that are convincing candidates for future studies. *GADD45A* (growth arrest and DNA-damage-inducible, alpha) is located on chromosome 1p31, a region with excess San ancestry in TB cases, and encodes a stress sensor protein that is involved in the regulation of myeloid cell innate immune function. Salerno et al. demonstrated that mice lacking Gadd45a are not able to recruit granulocytes and macrophages to the intraperitoneal cavity after administration of lipopolysaccharide (LPS) [15]. The recruitment of immune cells to the site of mycobacterial infection is crucial during TB. The oncostatin M gene (*OSM*) is also located in a region with excess San ancestry in TB cases (chromosome 22q12). Friendland et al. found that *M. tuberculosis* infection of monocytes resulted in prostaglandin-dependent OSM secretion, which synergized with tumour necrosis factor- *α* to drive fibroblast matrix metalloproteinases-1/-3 secretion [16].

Our analysis also found that chromosome 15q15 may contain an African TB susceptibility locus. Chromosome 15q was previously identified as containing TB susceptibility genes in a linkage study using African families [17] and fine-mapping localised the region to 15q11 [18]. Chromosome 17q22 also showed evidence of having excess African ancestry in TB cases. It is known that chromosome 17q11-q21 may contain a cluster of susceptibility genes for diseases caused by intramacrophage pathogens, such as *M. tuberculosis*[19]. Another gene with excess African TB ancestry was *B7-H5* (B7 homolog 5), located on chromosome 10q22. Its protein product plays a key regulatory role in T cell growth and cytokine production [20] and is expressed in macrophages.

Two of the 36 SNPs that were found to be moderately associated with having TB in a previous genome-wide association study (GWAS) are located in or proximal to regions identified in this study [9] (rs6694316 on 1p32 which is 16 cM way from the first SNP in the identified 1p31 region, that harbours the *GADD45A* gene, and rs4745272 on 9q21). Considering that the GWAS used the same SAC data set, we note that a number of new putative susceptibility regions were identified by our admixture mapping study, which would not have been evident based solely on single SNP association statistics.

### Study limitations

Due to the correlation between segments of ancestry, especially contiguous segments of ancestry, p-values that accurately quantify the statistical significance of our results could not be estimated in this study. Regions of excess ancestry in TB cases, having ancestry more than 2 standard deviations from the mean, were first identified. P-values were not estimated in this step due to the correlation between segments of ancestry. The identified regions would be the most probable regions to harbour ancestry-related TB susceptibility variants, and a statistical model was then used to determine which of the regions had excess ancestry in TB cases relative to controls. The estimated p-values from this model do not account for the fact that the variables were selected by first performing a case-only analysis. The statistical model also contained correlated variables, due to contiguous segments with similar ancestry. Correlation between a model’s independent variables may result in inflated variance, and estimated p-values may therefore be inflated (biased towards type II errors). We therefore note that the reported p-values should not be interpreted as a strict quantification of the statistical significance of the results, but that the p-values rather indicate which regions are most likely to harbour ancestry related TB susceptibility variants.

RFMix estimates of San ancestry were much lower than ADMIXTURE estimates. This may be explained by the small group size of our San source population as well as short tracts of San ancestry inherited via Southern African Bantu populations, and results in bias towards local ancestry deviations with lack of San ancestry. We speculate that roughly 50 San individuals would be required to alleviate the small group size problem (the RFMix authors used 30 reference individuals each from Native American, European and African populations in their simulations, but as the genetic distance between the San and Bantu populations is smaller than the genetic distances between the populations used by the RFMix authors, a larger number of reference individuals would be required to distinguish San and Bantu ancestry). We have worked around these limitations by focusing our attention on finding regions of excess San or excess San or Bantu ancestry, which are less likely to be affected by spurious deviation in local ancestry, and this approach also supports our hypothesis regarding the directionality of these ancestries and TB susceptibility. The possibility of short tracts of San ancestry harbouring TB susceptibility genes are also less likely compared to longer tracts, as the former are less likely to overlap in a group of individuals. Miss-identification of these short tracts as Bantu ancestry has therefore probably not resulted in loss of information regarding TB susceptibility.

The small size of the San source population also limited the number of ancestral chromosomes that were used in the generation of our simulated data set. As a result, the bias seen in the simulated local ancestry deviation distributions may have been exacerbated. Due to lack of accurate historical records, our simulation also did not take into account such complexities as the timing of admixture events, and potential inaccuracies of the source (reference) populations. Pasanuic et al. [21] recently evaluated the accuracy of multi-way LAI in a group of nuclear Latino families by determining whether the local ancestry of offspring is congruous with Mendelian inheritance (it is for example implausible that a child has European ancestry at a locus if neither parent has European ancestry at that locus). Multi-way LAI accuracy of several algorithms was shown to be much lower compared to reported accuracies calculated from simplified simulation data sets. Despite these issues, we have been able to use our simulated data set to demonstrate the relatively superior LAI accuracy of RFMix, and to explore the direction of potential local ancestry deviation bias.

Our SAC study group samples were genotyped with the aim of performing a case-only admixture mapping study, and as a result only a small number of controls were genotyped. As case-only admixture mapping has subsequently (and correctly) been described as inappropriate for multi-way admixed populations due to artefactual ancestry deviations arising from inaccuracies in LAI[22], we first identified regions of excess ancestry found in cases only, and then validated these findings by testing for excess ancestry in cases relative to controls. Despite the small number of controls that were available, bias in local ancestry inferences was still controlled, and a number of novel regions that contain highly plausible candidate TB susceptibility genes were uncovered by our study.

## Conclusion

The genetics of the South African Coloured population is arguably one of the most challenging and interesting examples found in present day multi-way admixed human populations. This is the first study to apply genome-wide admixture mapping to this highly complex group. We have demonstrated that admixture mapping is feasible in the South African Coloured population, a result which may be useful for other researchers that either study this population, or other populations with complex admixture. We have identified a number of novel candidate TB susceptibility genomic regions, as well as providing evidence to validate genetic loci previously implicated.

## Methods

### Sample collection and ethics approval

Individuals residing in the Cape Town suburbs of Ravensmead and Uitsig, and who self-identified as South African Coloured, were recruited to participate in this study. These suburbs have a low prevalence of HIV but a high incidence of TB, as well as a relatively homogenous socio-economic environment [7]. Bacterial confirmation (smear positive/culture positive) was used to identify TB patients. Healthy individuals with no prior history of TB were selected as controls. All the participants in this study were HIV negative. Our previous study of healthy children and young adults from the control community found that 80% of children older than 15 years had positive tuberculin skin tests (TST), an indication of latent infection with *M. tuberculosis*[23]. The majority of the control population is therefore TST positive, and with the average age of the controls in this study being 31 years, we estimate a TST positivity of 80% or above. These healthy individuals had no previous history of TB disease or treatment and were unrelated to all others included in the study.

This study was approved by the Ethics Committee of the Faculty of Health Sciences, Stellenbosch University (project registration numbers 95/072, NO6/07/132 and N11/07/210). Blood samples for DNA were collected with written informed consent. The research was conducted according to the principles expressed in the Declaration of Helsinki.

### Software

Web URLs, version information and parameter settings of the programs used in this study are summarized in Additional file 1: Table S3.

SHAPEIT [24] was used for phasing the SAC data set as well as the San source population data. A python script developed by J. Morrison was used to produce the ancestry break points of the simulated data set and to assign ancestry to segments along the chromosome. LAMP-LD [25] and RFMix [26] were used to infer local ancestry of the simulated data, whereas RFMix was used for inference of local ancestry of the SAC study group. ADMIXTURE [27] was used to estimate genome-wide ancestry of the SAC study group. PLINK [28] was used for merging admixed and source population data sets, in order to create input files required by ADMIXTURE. PLINK was also used to identify related individuals and filter SNPs according to quality control criteria. Prior to ADMIXTURE estimation and identification of related individuals, SNPs that were in LD were identified using PLINK and discarded, leaving 87 648 SNPs (see Additional file 1: Table S3 for PLINK parameter settings). Biofilter was used to identify genes that fall within specific regions of the genome. The R programming environment [29] was used for statistical analysis and the *ggplot2*[30] and *hexbin*[31] R packages was used to create the figures. The R *hierfstat* package was used to estimate *F*_*ST*_ (fixation index) between the source populations of the SAC, using the same data set that was created for ADMIXTURE estimation [32].

### Genotyping and quality control

969 individuals from the SAC study group were genotyped on the Affymetrix GeneChip Human Mapping 500K Array Set. After SNP calling [1], quality control and the removal of related individuals [9], the data set comprised 381 530 autosomal SNPs of 642 TB cases and 91 controls.

Source (a.k.a. reference) populations used to infer the ancestry of SAC individuals are summarized in Table 5 and the genetic distances (*F*_*ST*_) between these source populations are summarized in Additional file 1: Table S4. Populations used to represent the San were obtained from a private data access committee (contact corresponding author). The data set represents the same group analyzed by Schlebusch et al. [33], but was genotyped on the Affymetrix genotyping platform instead of the OmniExpress platform, which overlaps better with SNPs in the SAC study group data set. Phased HapMap3 Release 2 data was used to represent the remaining source populations. Pairwise IBS clustering was used to identify related individuals and only unrelated individuals (coefficient of relatedness < 0.5) were retained in the data sets. The San data set was filtered to remove SNPs that were not in Hardy-Weinberg equilibrium (p-value threshold of 0.0001) or had a large proportion of missing data (at least a 75% call rate).

**Table 5:** **Source population data**

Population	Group	Description	Source	Platform	Size
San		Ju |’hoansi San from North Namibia	Private	Affymetrix 6.0	21
Bantu	YRI	Yoruba in Ibadan, Nigeria	HapMap3	Release 2	112
Non-African	CEU	Utah residents with Northern and Western European ancestry, USA	HapMap3	Release 2	112
	GIH	Gujarati Indians from Houston, Texas, USA	HapMap3	Release 2	88
	JPT + CHB	Japanese in Tokyo, Japan and Han Chinese in Beijing, China	HapMap3	Release 2	170

Data sets used to represent the source populations of the South African Coloured population. The sample size reflects the group size after relative pairs have been removed.

### Phasing

As local ancestry inference requires phased input data, the SAC and San data sets were phased using a Markov model to estimate haplotypes, implemented in the SHAPEIT software. The genetic map used by the HapMap project to phase the HapMap data sets was utilized for phasing the SAC and San data sets (NCBI build 36 release 22, obtained from ftp://ftp.hapmap.org/hapmap/recombination/2008-03_rel22_B36/rates/). The genotypes of 733 SAC individuals were phased using 381 530 autosomal SNPs. The San data set of 21 individuals were phased using 866 382 autosomal SNPs.

### Combining data sets

After quality control and phasing of the SAC and San source population data sets, the data sets were reduced to contain only those SNPs present in all the data sets (328 866 autosomal SNPs). Where required, strand and reference alleles of the SAC and San data sets were flipped to match the HapMap data sets. The centimorgan (cM) genomic positions used by PCAdmix and RFMix to determine ancestry windows were calculated using the NCBI build 36 release 22 genomic map (obtained from ftp://ftp.hapmap.org/hapmap/recombination/2008-03_rel22_B36/rates/). The base pair positions of SNPs were obtained from the HapMap data, and in the case where an exact base pair position match was not found in the genomic map, the base pair position of the SNP was converted to cM by using a weighted average of the cM positions of the two SNPs closest to it.

### Simulation

Using an approach similar to that of Pasanuic et al. [21] and Price et al. [34], the ancestry breakpoints of 1500 admixed chromosomes (chromosome 1) were first generated. Recombination positions and thus breaks in ancestry on each chromosome were generated using a random walk from base pair position zero to the end of the chromosome, with ancestry crossovers occurring as a Poisson process. The rate of the Poission process was set to 10 (the assumed number of generations since admixture) times a recombination rate of 10^-8^. The average number of breakpoints per chromosome was 35. After determining the breakpoints, segments of ancestry on each chromosome were assigned as San, YRI, CEU, GIH and JPT + CHB using proportions 0.33, 0.28, 0.19, 0.13 and 0.07 respectively, corresponding to the genome-wide ancestry estimates of Chimusa et al. [9]. The ancestry assignments were based on draws from a uniform (0, 1) distribution, and determining whether a draw falls between the interval [0.00, 0.33) (San), [0.33, 0.61) (YRI), [0.61, 0.80) (CEU), [0.80, 0.93) and [0.93, 1] (JPT + CHB).

Segments of ancestry assigned in this manner were then used to construct 1500 admixed chromosomes (chromosome 1). 10 source population chromosomes were selected randomly from each of the source data sets. The remaining chromosomes were set aside to use as input source populations for LAI. Admixed chromosomes were constructed by randomly copying segments of ancestry from the selected source population chromosomes, corresponding to the ancestry assigned to the segment. As a simple example, consider an admixed chromosome with San ancestry for SNPs at positions 1 to 100 on the chromosome, and CEU ancestry for SNPs at positions 101 to 200. The admixed chromosome would be constructed by randomly selecting a San chromosome and copying the SNPs at positions 1 to 100, followed by randomly selecting a CEU chromosome and copying the SNPs at positions 101 to 200.

Note that a limited number of source population chromosomes were used to simulate the admixed chromosomes. As a result, the data set does not contain independent observations. Unlike most LAI algorithms, the global ancestry proportion estimation algorithm implemented in the software program ADMIXTURE assumes independent observations. Taken together with the limited number of SNPs that are available compared to genome-wide data (only chromosome 1 SNPs are available), the simulated chromosome 1 data set is not suited to the estimation of chromosome-wide ancestry using ADMIXTURE, and RFMix chromosome-wide estimates were used instead.

### Labelling ancestry

LAMP-LD, RFMix and ADMIXTURE were run using five source populations (Table 5), but since only San and Bantu genome-wide ancestry is independently associated with TB susceptibility [8, 9], European, South Asian and East Asian ancestries were merged and labelled as non-African after inference was performed.

### Delineating called ancestry segments

RFMix labels the called ancestry of each SNP along a chromosome and does not identify windows of ancestry across a chromosome in its output. Segments of ancestry were therefore identified by determining the SNP positions where a switch in ancestry occurs for at least one chromosome. Each ancestry segment starts at such a position, and ends one SNP before the next ancestry switch position.

### Calculating genome-wide ancestry using local ancestry

The genome-wide ancestry of each of the 733 SAC individuals was calculated using local ancestry called by RFMix, as follows: The number of SNPs labeled as a particular ancestry was counted per individual for each of the individual’s 22 × 2 chromosomes. This total count was divided by the total number of SNPs across the genome, yielding an estimate for the individual’s genome-wide ancestry.

### Calculating local ancestry deviation

After delineating the called ancestry of the 1500 simulated chromosomes into 1077 segments, the mean San, Bantu and non-African ancestry of each segment was calculated. The local ancestry deviation of each segment was then calculated, seperately for each of the three ancestries, by subtracting the overall RFMix mean ancestry from the mean ancestry of the segment.

In the same way, after delineating the called ancestry of 733×2×22 chromosomes in the SAC study group into 13 860 segments, the mean ancestry of each segment was calculated for each of the three source ancestries. The local ancestry deviation of each segment was calculated by subtracting the overall RFMix mean genome-wide ancestry from the mean ancestry of the segment, for each of the ancestries.

### Correlation between miss-called ancestry and deviation in ancestry in simulated data

After local ancestry deviations were calculated for each of the segments identified in the simulated data set, and segments with miss-called ancestry were identified by comparing ancestry called by RFMix to the known ancestry of each segment, the number of segments with miss-called ancestries were calculated per identified segment. This was done for each of the six possible pairs of miss-called ancestry (San miss-called as Bantu, San miss-called as non-African, Bantu miss-called as San, Bantu miss-called as non-African, non-African miss-called as San and non-African miss-called as Bantu). A large number of miss-calls occurred in the segment of ancestry that spans the centromere, with a standardized local ancestry deviation Z-value of -0.7109, 0.7052, and 0.1518 for San, Bantu and non-African ancestry respectively. After discarding this outlying segment, Pearson’s correlation coefficient was calculated between the number of miss-called segments and local ancestry deviation, for each of the six possible pairs of miss-called ancestry and San, Bantu and non-African local ancestry deviation combinations.

### Calculating ancestry inference accuracy using simulated data

Accuracy of LAI using the simulated data set was calculated per chromosome as follows. The ancestry assigned to each SNP in the data set by the simulation process was compared to the ancestry assigned to the SNP by LAI. The proportion of SNPs that the LAI correctly assigned to each chromosome was then calculated. For each of the three considered ancestries (San, Bantu and non-African), the proportion of SNPs that were miss-called for each of the other two ancestries was also calculated. The miss-called proportions were calculated across all SNPs on the chromosome, as well as per number of SNPs of that particular ancestry.

### Relationships between miss-called ancestry, tract length and degree of admixture

The lengths of tracts of ancestry in the simulated data set were calculated in terms of the number of SNPs that constitute a track. Each track’s corresponding proportion of miss-called SNPs were calculated by comparing the ancestry assigned by RFMix to each SNP with the known ancestry of the SNP, and then dividing the number of miss-called SNPs by the length of the tract. Pearson’s correlation coefficient was calculated to quantify the relationship between the length of a track and the proportion of errors on a track.

The number of tracks of ancestry present in each simulated chromosome was counted, and was used to represent a chromosome’s degree of admixture. The number of miss-called SNPs was counted per chromosome, by comparing the ancestry assigned by RFMix to each SNP with the known ancestry of the SNP. Pearson’s correlation coefficient was calculated to quantify the relationship between the number of tracks of ancestry of a chromosome and the number of miss-called SNPs on a chromosome.

### Statistical analyses

A single linear mixed-effects model was used to compare the number of SNPs that were miss-called by LAMP-LD vs. RFMix in the simulated data set. The proportion of SNPs that were miss-called for a particular pair of ancestries (the proportion of San SNPs miss-called as Bantu, the proportion of San SNPs miss-called as non-African, etc.) was log transformed for analysis, since the proportions were positively skewed. The interaction between software program (LAMP-LD or RFMix) and pair of ancestries was tested, while chromosome identifiers were specified as random effect, in order to adjust for the correlation between between different miss-call proportions on the same chromosome.

Two joint logistic regression models were used to test whether San and African ancestry differs between TB cases and controls (the statistical modelling term “joint” means that all the effects were estimated jointly in a single model, instead of doing an individual test for each combination of ancestry and segment). Joint modelling has the advantage of providing estimates that are adjusted for all other predictors in the same model. Case-control status was specified as outcome variable and the interaction of segment identifier and presence or absence of the particular ancestry (San/not-San or African/not-African) was specified as predictor. This statistical interaction between a specific ancestry and a segment identifier can be described as a predictor combining an ancestry indicator with the segment identifier. That means that the odds ratio for each segment is the estimated odds of having TB versus not having TB for a San segment, compared to the odds for a non-San segment, and the interpretation is analogous for African ancestry. Since age, gender and genome-wide ancestry distributions differ between TB cases and controls, the models were also adjusted for these variables.

## Electronic supplementary material

Additional file 1:**Supplementary Figures and Tables.** 10 figures and 4 tables are found in this file. (PDF 298 KB)

## References

[1] de Wit E, Delport W, Rugamika CE, Meintjes A, Möller M, van Helden PD, Seoighe C, Hoal EG (2010). **Genome-wide analysis of the structure of the South African Coloured Population in the Western Cape**. Hum Genet.

[2] Chimusa ER, Daya M, Möller M, Ramesar R, Henn BM, van Helden PD, Mulder NJ, Hoal EG (2013). **Determining ancestry proportions in complex admixture scenarios in South Africa using a novel proxy ancestry selection method**. PLoS ONE.

[3] Tishkoff SA, Reed FA, Friedlaender FR, Ehret C, Ranciaro A, Froment A, Hirbo JB, Awomoyi AA, Bodo J-M, Doumbo O (2009). **The genetic structure and history of Africans and African Americans**. Science.

[4] Patterson N, Petersen DC, Van Der Ross RE, Sudoyo H, Glashoff RH, Marzuki S, Reich D, Hayes VM (2010). **Genetic structure of a unique admixed population: implications for medical research**. Hum Mol Genet.

[5] Quintana-Murci L, Harmant C, Quach H, Balanovsky O, Zaporozhchenko V, Bormans C, van Helden PD, Hoal EG, Behar DM (2010). **Strong maternal Khoisan contribution to the South African coloured population: a case of gender-biased admixture**. Am J Hum Genet.

[6] Daya M, van der Merwe L, Galal U, Möller M, Salie M, Chimusa ER, Galanter JM, van Helden PD, Henn BM, Gignoux CR (2013). **A panel of ancestry informative markers for the complex five-way admixed South African Coloured population**. PLoS ONE.

[7] Barreiro LB, Neyrolles O, Babb CL, Tailleux L, Quach H, McElreavey K, Van Helden PD, Hoal EG, Gicquel B, Quintana-Murci L (2006). **Promoter variation in the DC-SIGN-encoding gene CD209 is associated with tuberculosis**. PLoS Med.

[8] Daya M, van der Merwe L, van Helden PD, Möller M, Hoal EG (2014). **The role of ancestry in TB susceptibility of an admixed South African population**. Tuberculosis.

[9] Chimusa ER, Zaitlen N, Daya M, Möller M, van Helden PD, Mulder NJ, Price AL, Hoal EG (2013). **Genome-wide association study of ancestry-specific TB risk in the South African coloured population**. Hum Mol Genet.

[10] Winkler CA, Nelson GW, Smith MW (2010). **Admixture mapping comes of age**. Annu Rev Genomics Hum Genet.

[11] Van der Spuy GD, Kremer K, Ndabambi SL, Beyers N, Dunbar R, Marais BJ, van Helden PD, Warren RM (2009). **Changing*****Mycobacterium tuberculosis*****, population highlights clade-specific pathogenic characteristics**. Tuberculosis.

[12] Dubos RJ, Dubos J (1952). The White Plague: Tuberculosis, Man, and Society.

[13] Nurse GT, Weiner JS, Jenkins T (1985). The peoples of Southern Africa and their affinities.

[14] Gravel S (2012). **Population genetics models of local ancestry**. Genetics.

[15] Salerno DM, Tront JS, Hoffman B, Liebermann DA (2012). **Gadd45a and gadd45b modulate innate immune functions of granulocytes and macrophages by differential regulation of p38 and JNK signaling**. J Cell Physiol.

[16] O’Kane CM, Elkington PT, Friedland JS (2008). **Monocyte-dependent oncostatin m and TNF-alpha synergize to stimulate unopposed matrix metalloproteinase-1/3 secretion from human lung fibroblasts in tuberculosis**. Eur J Immunol.

[17] Bellamy R, Beyers N, McAdam KP, Ruwende C, Gie R, Samaai P, Bester D, Meyer M, Corrah T, Collin M, Camidge DR, Wilkinson D, Hoal-Van Helden E, Whittle HC, Amos W, van Helden P, Hill AV (2000). **Genetic susceptibility to tuberculosis in Africans: a genome-wide scan**. Proc Natl Acad Sci.

[18] Cervino AC, Lakiss S, Sow O, Bellamy R, Beyers N, Hoal-Van Helden E, van Helden P, McAdam KP, Hill AV (2002). **Fine mapping of a putative tuberculosis-susceptibility locus on chromosome 15q11-13 in African families**. Hum Mol Genet.

[19] Möller M, Nebel A, Valentonyte R, van Helden PD, Schreiber S, Hoal EG (2009). **Investigation of chromosome 17 candidate genes in susceptibility to TB in a South African population**. Tuberculosis.

[20] Zhu Y, Yao S, Iliopoulou BP, Han X, Augustine MM, Xu H, Phennicie RT, Flies SJ, Broadwater M, Ruff W (2013). **B7-H5 costimulates human T cells via CD28H**. Nat Commun.

[21] Pasaniuc B, Sankararaman S, Torgerson DG, Gignoux C, Zaitlen N, Eng C, Rodriguez-Cintron W, Chapela R, Ford JG, Avila PC (2013). **Analysis of Latino populations from GALA and MEC studies reveals genomic loci with biased local ancestry estimation**. Bioinformatics.

[22] Seldin M, Pasaniuc B, Price A (2011). **New approaches to disease mapping in admixed populations**. Nat Rev Genet.

[23] Gallant CJ, Cobat A, Simkin L, Black GF, Stanley K, Hughes J, Doherty TM, Hanekom WA, Eley B, Beyers N, Jaïs JP, van Helden P, Abel L, Alcaïs A, Hoal EG, Schurr E (2010). **Impact of age and sex on mycobacterial immunity in an area of high tuberculosis incidence**. Int J Tuberc Lung Dis.

[24] Delaneau O, Zagury J-F, Marchini J (2013). **Improved whole-chromosome phasing for disease and population genetic studies**. Nat Methods.

[25] Baran Y, Pasaniuc B, Sankararaman S, Torgerson DG, Gignoux C, Eng C, Rodriguez-Cintron W, Chapela R, Ford JG, Avila PC (2012). **Fast and accurate inference of local ancestry in Latino populations**. Bioinformatics.

[26] Maples BK, Gravel S, Kenny EE, Bustamante CD (2013). **RFMix: a discriminative modeling approach for rapid and robust local-ancestry inference**. Am J Hum Genet.

[27] Alexander D, Novembre J, Lange K (2009). **Fast model-based estimation of ancestry in unrelated individuals**. Genome Res.

[28] Purcell S, Neale B, Todd-Brown K, Thomas L, Ferreira MAR, Bender D, Maller J, Sklar P, de Bakker PIW, Sham PC, Daly M J (2007). **PLINK: a tool set for whole-genome association and population-based linkage analyses**. Am J Hum Genet.

[29] R Core Team (2013). R: A Language and Environment for Statistical Computing.

[30] Wickham H (2009). Ggplot2: Elegant Graphics for Data Analysis.

[31] Carr D, ported by Nicholas Lewin-Koh, Maechler M, contains copies of lattice function written by Deepayan Sarkar (2014). **Hexbin: Hexagonal Binning Routines**.

[32] Goudet J (2013). **Hierfstat: Estimation and Tests of Hierarchical F-statistics**.

[33] Schlebusch CM, Skoglund P, Sjödin P, Gattepaille LM, Hernandez D, Jay F, Li S, De Jongh M, Singleton A, Blum MGB (2012). **Genomic variation in seven Khoe-San groups reveals adaptation and complex African history**. Science.

[34] Price AL, Tandon A, Patterson N, Barnes KC, Rafaels N, Ruczinski I, Beaty TH, Mathias R, Reich D, Myers S (2009). **Sensitive detection of chromosomal segments of distinct ancestry in admixed populations**. PLoS Genet.

