# Supplementary material for: Using multi-way admixture mapping to elucidate TB susceptibility in the South African Coloured population
Source: BMC Genomics. 2014 Nov 25;15(1):1021. doi: 10.1186/1471-2164-15-1021 (PMC4256931; doi:10.1186/1471-2164-15-1021)
Supplement: Supplementary file 1 — Additional file 1:Supplementary Figures and Tables. 10 figures and 4 tables are found in this file. (PDF 298 KB) [file 12864_2014_6716_MOESM1_ESM.pdf]

Supplementary Figures

Figure S1 Difference between ancestry called by RFMix and known ancestry per individual. The known ancestry of a simulated data set of 750 SAC individuals is compared to the ancestry calledy by RFMix per individual (chromosome 1). Histograms of the difference between the called mean ancestry and known mean ancestry of each individual are shown, per each of the three source ancestries.

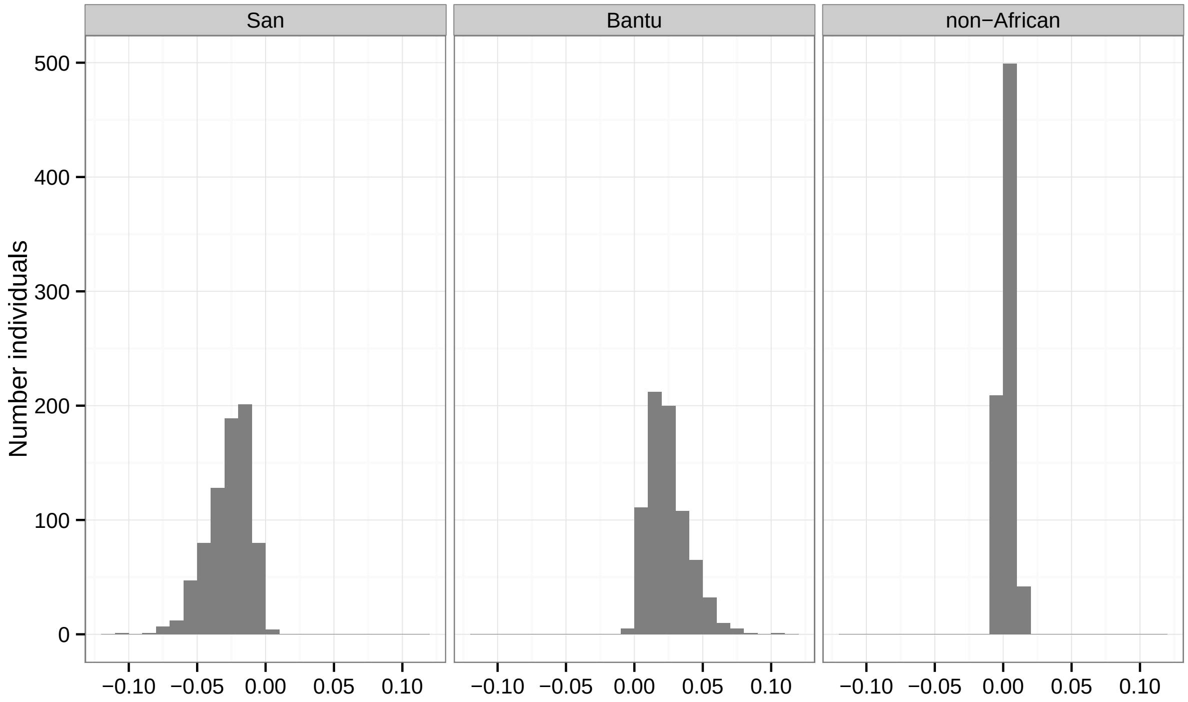

Figure S2 Scatterplots of the number of miss-called ancestry segments against deviation in ancestry in simulated data. Miss-called ancestry was identified by comparing the known ancestry of a simulated data set of 1500 SAC chromosomes to the ancestry called by RFMix (chromosome 1). Deviations in ancestry were calculated by subtracting the overall RFMix mean ancestry from the local mean ancestry of each segment.

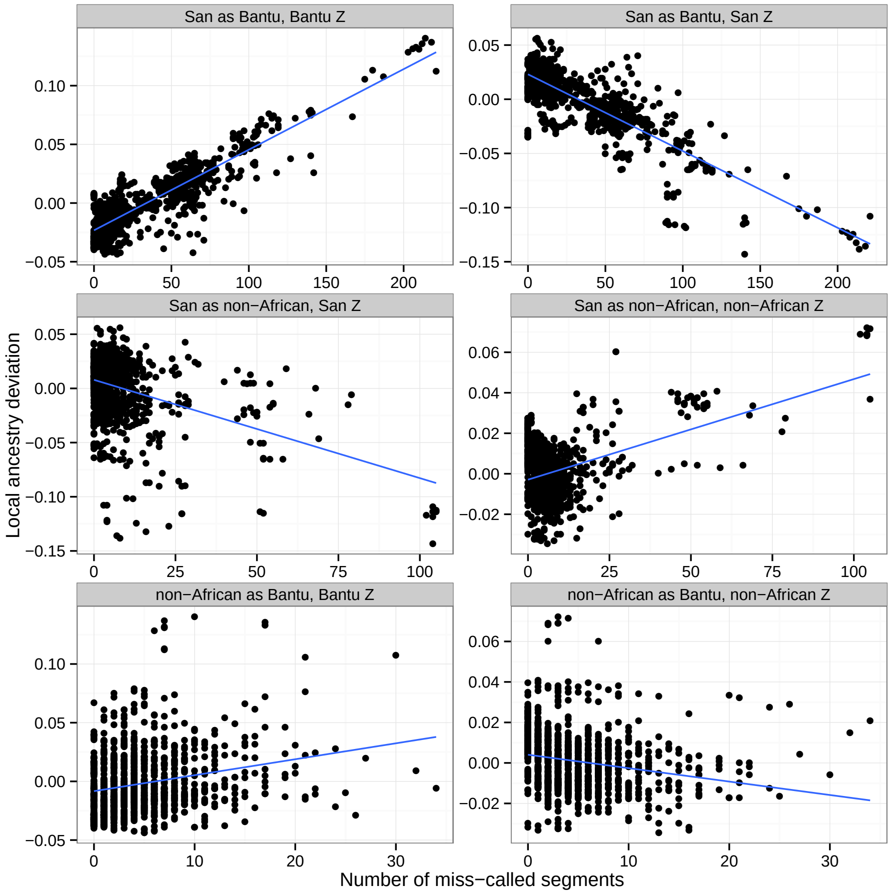

Figure S3 Local ancestry deviations in simulated data. Histograms of local ancestry deviations in the simulated data set are shown in this figure, for each of the source ancestries. The deviation of each segment was calculated by subtracting the overall RFMix mean ancestry from the local mean ancestry of the segment (chromosome 1). Standardized deviation scores are shown at the bottom of the horizontal axis.

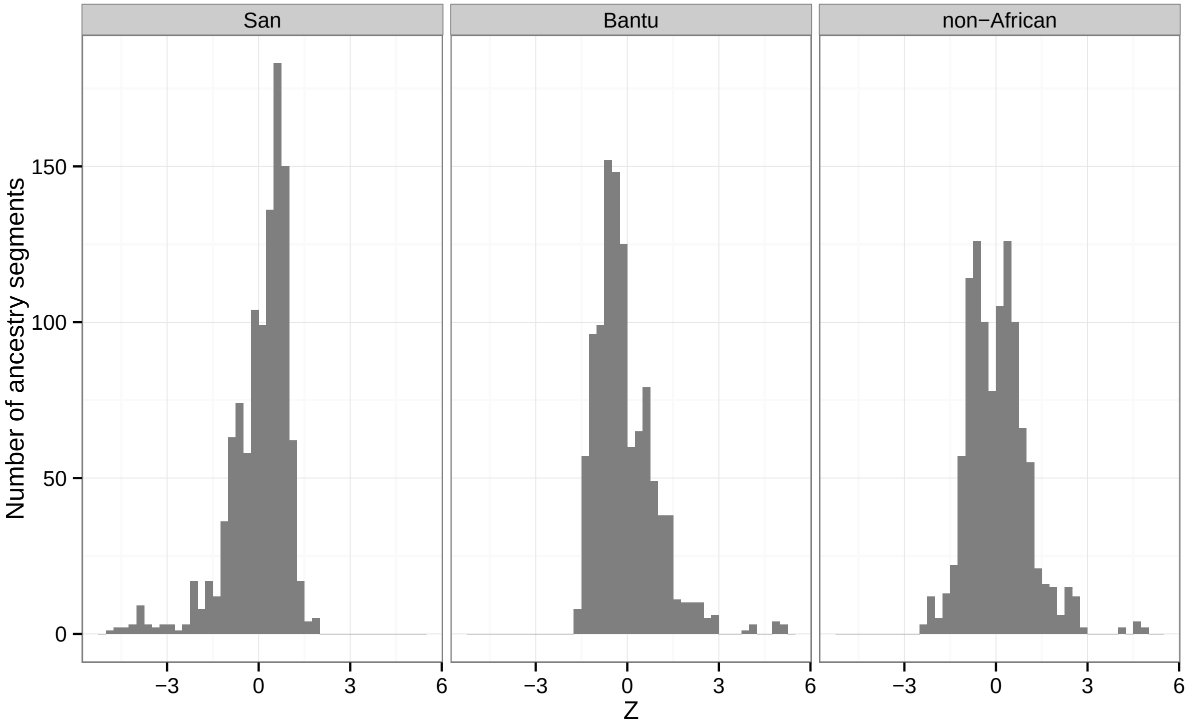

Figure S4 Distribution of miss-called San ancestry segments in simulated data. The figure shows the base pair positions of San ancestry segments that were miss-called by RFmix to have Bantu or non-African ancestry, and the number of segments that were miss-called at a position, in a simulated data set of 1500 SAC haplotypes (chromosome 1). Data points are shaded according to deviation from the RFMix overall mean San ancestry, where darker shades indicate lower San ancestry compared to the mean.

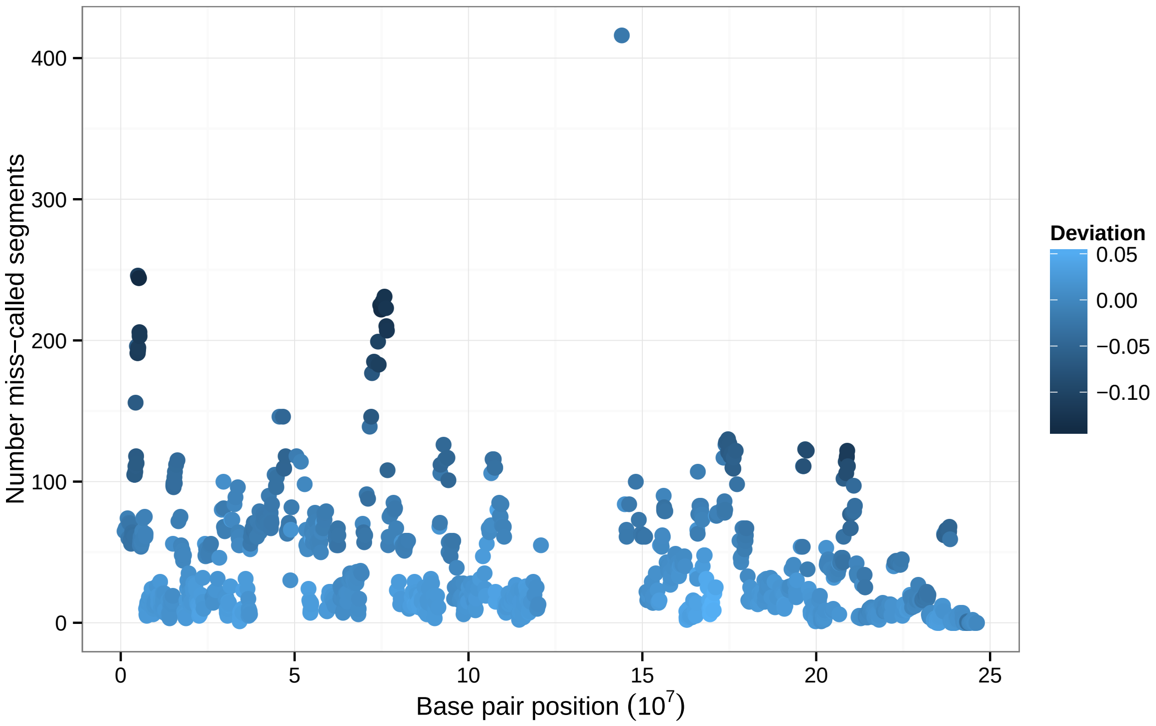

Figure S5 Distribution of the length of tracts of ancestry and the proportion of SNPs with miss-called ancestry per tract in the simulated data. The lengths of tracts of ancestry in a simulated data set of 1500 SAC chromosomes (chromosome 1) were calculated in terms of the number of SNPs that constitute a track, and are shown on the x-axis. The proportion of SNPs that were miss-called were calculated per track by comparing the ancestry assigned by RFMix to each SNP with the known ancestry of the SNP, and is shown on the y-axis (number miss-called SNPs divided by the length of the tract). Hexagons denote one or more observations; the darker the shading, the more observations are represented.

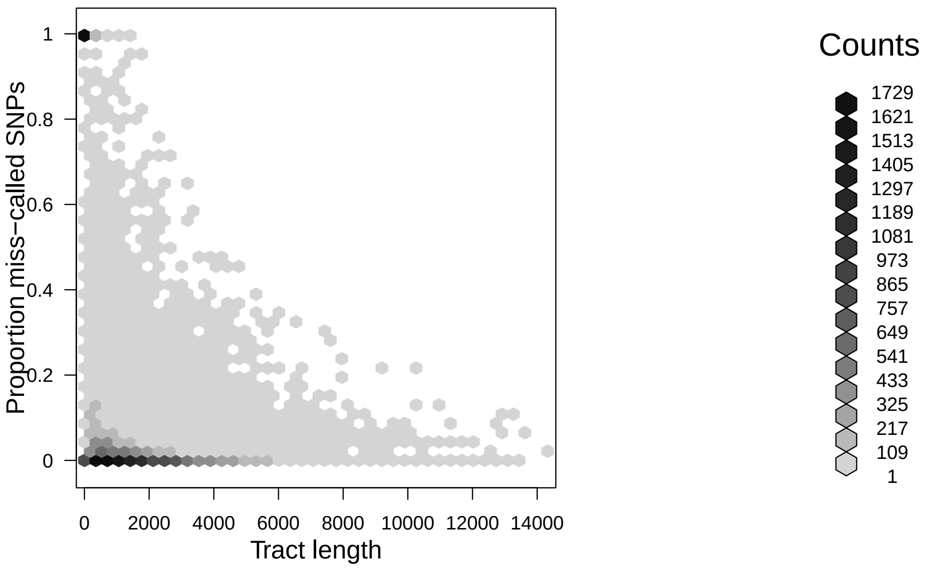

Figure S6 Scatterplot of the number of tracts of ancestry on a chromosome and the number of miss-called SNPs for that chromosome. The number of tracts of ancestry in a simulated data set of 1500 SAC chromosomes (chromosome 1) were counted per chromosome and is shown on the x-axis. The corresponding number of miss-called SNPs for each simulated chromosome was determined by comparing the ancestry assigned by RFMix to each SNP with the known ancestry of the SNP, and is shown on the y-axis. Each data point therefore represents a single simulated chromosome, with its number of ancestry tracts read from the x-axis, and its number of miss-called SNPs read from the y-axis.

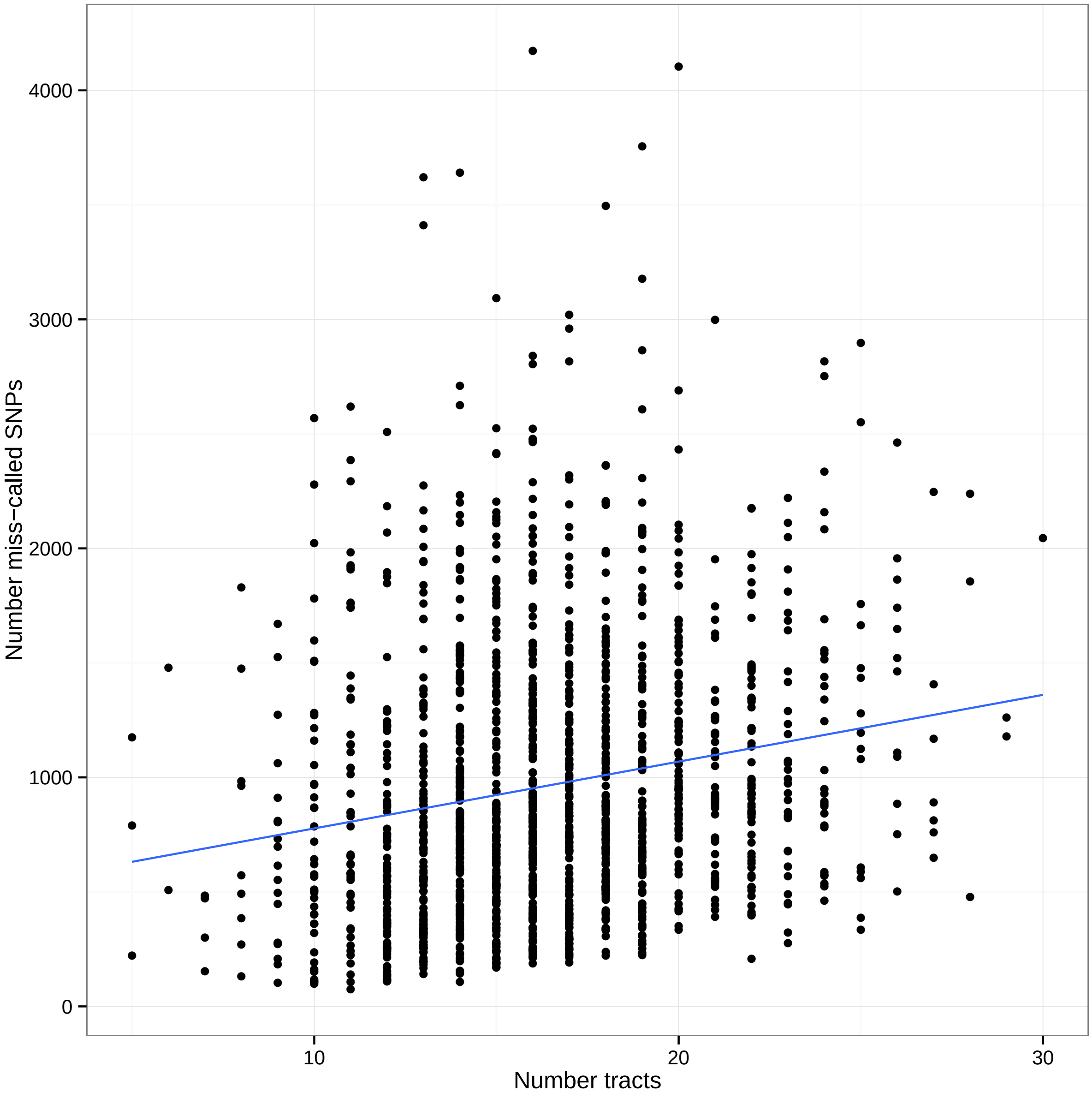

Figure S7 Difference between RFMix and ADMIXTURE estimates of genome-wide ancestry in the SAC study group. The difference between the genome-wide ancestries estimated by RFMix and ADMIXTURE in a study group of 733 SAC individuals are shown in this figure. Histograms of the difference between each individual's RFMix and ADMIXTURE ancestry estimate are shown, per each of the three source ancestries.

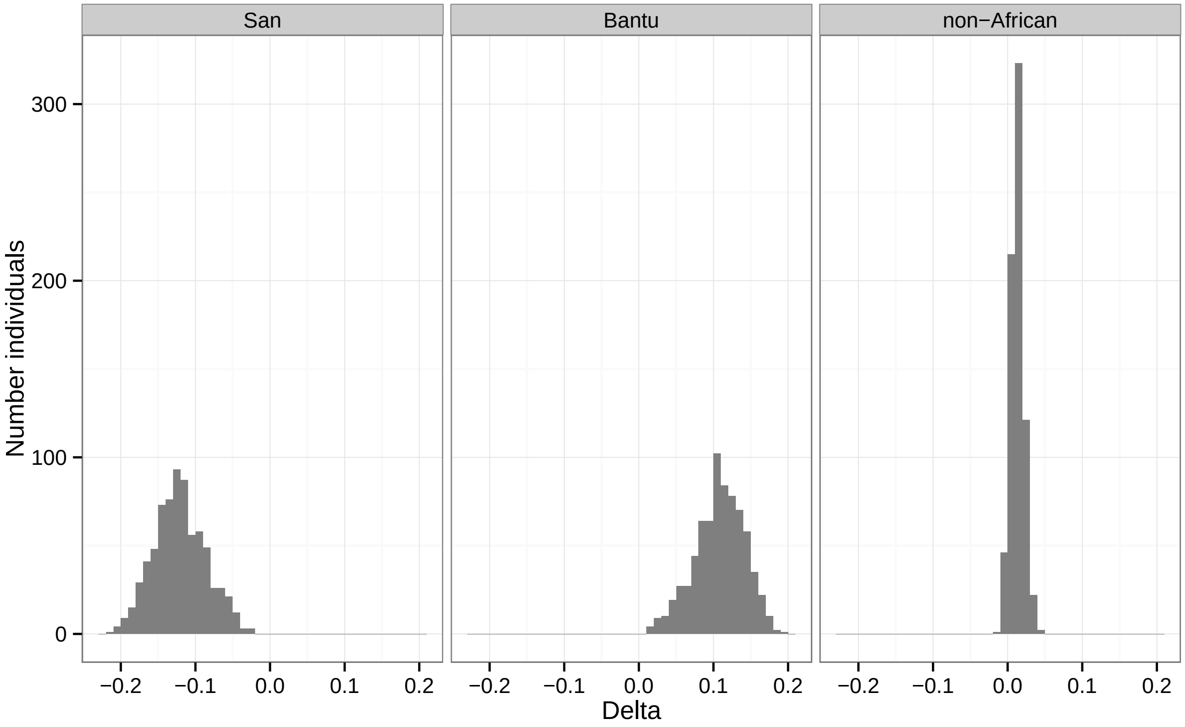

Figure S8 Boxplots of ancestry tract lengths in the SAC study group. The distribution of the mean San, Bantu and European tract lengths of each of the 733 individuals in the SAC study group are depicted in this figure.

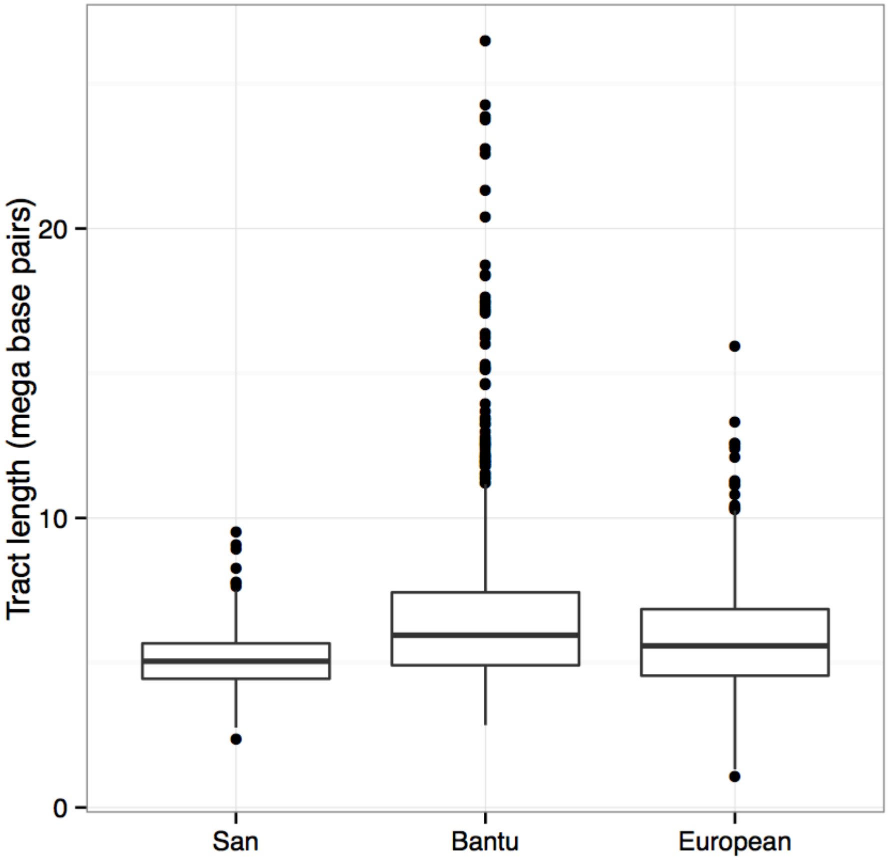

Figure S9 Histograms of local ancestry deviations in the SAC study group. Histograms of the deviations of local ancestry in the SAC study group (642 TB cases and 91 controls) are shown in this figure, for each of the source ancestries. The deviation of each segment was calculated by subtracting the mean RFMix genome-wide ancestry from the mean local ancestry of the segment, seperately for cases and controls. Standardized deviation scores are shown at the bottom of the horizontal axis.

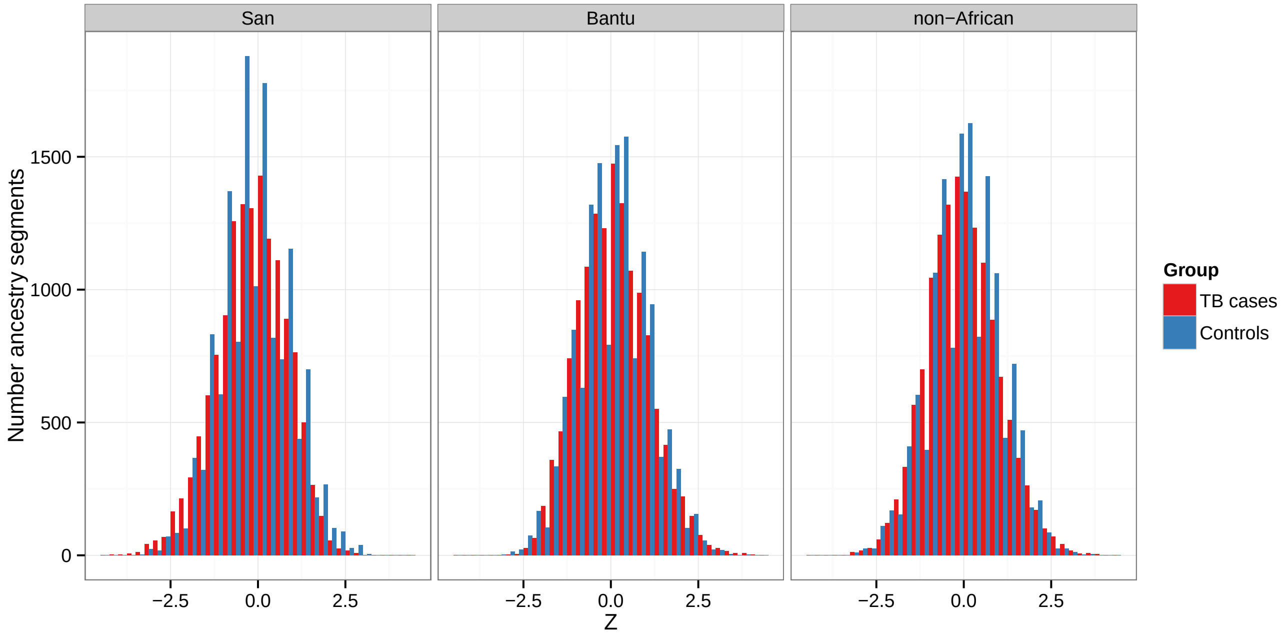

Figure S10 Boxplots of local ancestry deviations in the SAC study group. Boxplots of the standardized deviations of local ancestry in the SAC study group (642 TB cases and 91 controls) are shown in this figure, for each of the source ancestries. The deviation of each segment was calculated by subtracting the mean RFMix genome-wide ancestry from the mean local ancestry of the segment, separately for cases and controls. The local ancestry deviations were then standardized by dividing by the standard deviation of the local ancestry deviations.

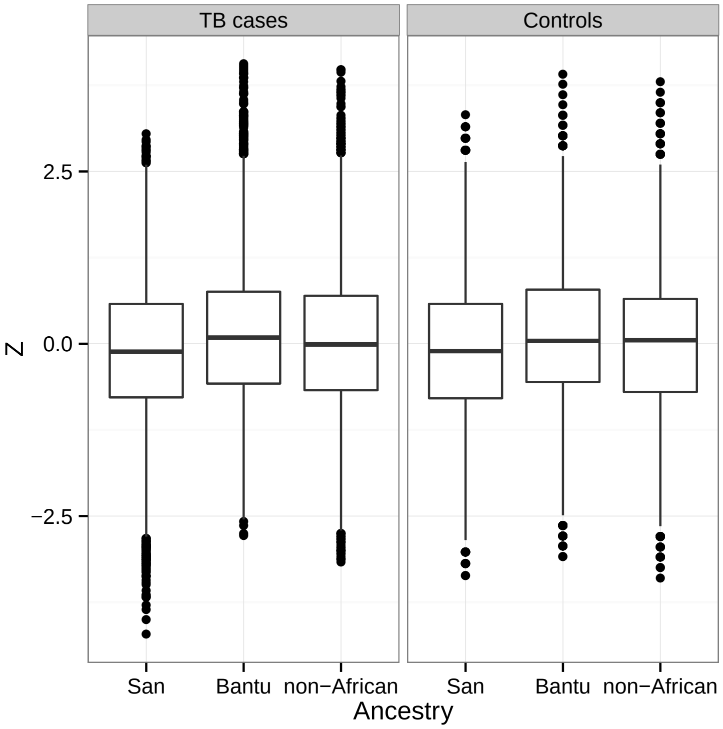

Supplementary Tables

Table S1 Statistical significance of regions of the genome with excess San ancestry in TB cases relative to controls. This table summarizes regions of the genome with excess San ancestry, found in TB cases relative to controls, after adjusting for age, gender and genome-wide San ancestry. Segments were labeled according to their position on the chromosome; contiguous segments of ancestry therefore have contiguous segment identifiers.

Region	Segment ID	Begin-end SNP	Length (Nr SNPs)	Mean San ancestry TB Cases	Controls	P-value
1p31	375	rs12144711-rs10789239	107674 (24)	0.2897	0.1995	0.0135
1p31	376	rs4655567-rs4548410	254010 (44)	0.2928	0.2160	0.0326
1p31	377	rs12025677-rs11209202	131840 (20)	0.2936	0.2160	0.0319
1p31	378	rs10889741-rs6691251	88184 (9)	0.2889	0.2105	0.0269
1p31	379	rs2566762-rs7554551	82567 (26)	0.2858	0.1940	0.0099
5p13	114	rs10513153-rs1445823	130346 (35)	0.2827	0.2160	0.0238
5p13	235	rs16904004-rs6870368	115695 (17)	0.2843	0.2160	0.0465
9q21	269	rs2309428-rs6559488	131678 (20)	0.2858	0.2050	0.0231
9q21	270	rs11138342-rs11139997	353460 (40)	0.2889	0.2105	0.0319
9q21	271	rs10511968-rs11140836	172263 (28)	0.2850	0.2050	0.0294
9q21	272	rs11140862-rs7875663	573992 (84)	0.2967	0.2050	0.0138
9q21	273	rs6560137-rs7350298	302822 (55)	0.2952	0.2050	0.0203
9q21	274	rs1028879-rs7041925	179239 (37)	0.2913	0.2105	0.0339
9q21	275	rs2909293-rs1847503	332682 (59)	0.2936	0.2050	0.0222
22q12	93	rs16986925-rs5762996	143883 (42)	0.2882	0.2215	0.0326
22q12	94	rs132275-rs2301290	135145 (10)	0.2874	0.2215	0.0358
22q12	96	rs2857641-rs6006426	612310 (65)	0.2827	0.2215	0.0355

Table S2 Statistical significance of regions of the genome with excess African (San or Bantu) ancestry in TB cases relative to controls. This table summarizes regions of the genome with excess African ancestry, found in TB cases relative to controls, after adjusting for age, gender and genome-wide African ancestry. Segments were labeled according to their position on the chromosome; contiguous segments of ancestry therefore have contiguous segment identifiers.

			Length	Mean San ancestry		Mean Bantu ancestry		
Region	Segment ID	Begin-end SNP	(Nr SNPs)	TB Cases	Controls	TB Cases	Controls	P-value
5q11	244	rs1450660-rs1822824	303696 (33)	0.2702	0.2105	0.3770	0.3423	0.0081
5q11	250	rs26090-rs1382907	739064 (70)	0.2726	0.1885	0.3754	0.3588	0.0049
6q15	402	rs11969733-rs285612	217975 (24)	0.2375	0.2050	0.4104	0.3478	0.0091
6q15	403	rs16882779-rs790604	24779 (5)	0.2375	0.2050	0.4112	0.3478	0.0087
10q22	368	rs827299-rs1338638	57072 (21)	0.2298	0.1995	0.4361	0.3972	0.0351
10q22	369	rs1338637-rs12264572	94894 (12)	0.2227	0.1995	0.4424	0.3917	0.0223
10q22	370	rs7076330-rs10999736	138544 (22)	0.2196	0.2050	0.4439	0.3863	0.0297
10q22	371	rs16928536-rs3740458	63196 (16)	0.2204	0.2050	0.4463	0.3753	0.0126
10q22	372	rs7075861-rs1417207	95418 (14)	0.2196	0.2215	0.4463	0.3643	0.0190
10q22	373	rs10999804-rs2394797	57794 (12)	0.2243	0.2325	0.4439	0.3588	0.0189
10q22	374	rs7088556-rs17634834	32303 (4)	0.2266	0.2380	0.4416	0.3533	0.0189
10q22	376	rs10509336-rs7094749	111700 (19)	0.2562	0.2215	0.4073	0.3698	0.0222
10q22	377	rs10999960-rs9415039	64778 (6)	0.2656	0.2325	0.4027	0.3698	0.0310
10q22	378	rs10762477-rs7090957	176032 (23)	0.2695	0.2325	0.3964	0.3698	0.0309
10q22	379	rs10509339-rs10509767	338118 (38)	0.2625	0.2380	0.3988	0.3643	0.0314
10q22	380	rs10762505-rs3740293	1359930 (71)	0.2648	0.2380	0.3972	0.3698	0.0346
10q22	381	rs1004059-rs11000831	327517 (17)	0.2625	0.2215	0.3964	0.3698	0.0125
10q22	382	rs10824049-rs10824259	1080040 (72)	0.2609	0.2270	0.4003	0.3643	0.0094
10q22	383	rs10762651-rs7088635	321064 (29)	0.2601	0.2160	0.4050	0.3753	0.0086
10q22	384	rs4612741-rs2133705	336875 (35)	0.2570	0.2160	0.4089	0.3753	0.0080
10q22	385	rs1124372-rs9415136	131915 (31)	0.2531	0.2105	0.4097	0.3753	0.0070
10q22	386	rs4746341-rs17445672	222280 (41)	0.2555	0.2050	0.3972	0.3753	0.0110
10q22	387	rs16932945-rs1992012	126121 (23)	0.2586	0.1995	0.3902	0.3643	0.0038
10q22	388	rs17376389-rs2637266	276265 (75)	0.2625	0.1940	0.3847	0.3753	0.0068
10q22	389	rs1907323-rs4980117	360363 (60)	0.2632	0.1940	0.3863	0.3698	0.0033
10q22	391	rs2395453-rs7083934	200022 (30)	0.2508	0.1940	0.3964	0.3863	0.0162
10q22	395	rs1877998-rs11815134	113578 (12)	0.2702	0.2380	0.3863	0.3643	0.0410
10q25	508	rs3014204-rs17115877	237854 (22)	0.2562	0.1940	0.3910	0.3533	0.0042
10q25	514	rs10884128-rs10509806	507163 (60)	0.2702	0.2160	0.3777	0.3478	0.0213
10q25	542	rs10506868-rs11196030	82164 (14)	0.2586	0.2050	0.3972	0.3808	0.0370
15q15	136	rs1712435-rs677845	110381 (21)	0.2305	0.2435	0.4229	0.3423	0.0289
15q15	137	rs588695-rs493177	1088795 (90)	0.2290	0.2270	0.4221	0.3533	0.0293
15q15	138	rs574065-rs16966424	1455279 (71)	0.2274	0.2270	0.4213	0.3533	0.0341
17q22	296	rs7210845-rs9894332	55957 (9)	0.2648	0.2105	0.3847	0.3313	0.0082
17q22	297	rs17759236-rs9891519	269818 (44)	0.2765	0.2160	0.3832	0.3368	0.0090
17q22	298	rs929585-rs7208587	160574 (28)	0.2741	0.2160	0.3832	0.3368	0.0101
17q22	299	rs17760268-rs4793823	128914 (29)	0.2757	0.2105	0.3793	0.3423	0.0118
17q22	300	rs10491158-rs8069500	74109 (18)	0.2819	0.2270	0.3793	0.3203	0.0038
17q22	301	rs3914804-rs17820808	23232 (7)	0.2827	0.2270	0.3793	0.3203	0.0034
17q22	302	rs4794665-rs2525997	120913 (16)	0.2780	0.2270	0.3855	0.3148	0.0015
17q22	303	rs205499-rs11079268	128522 (9)	0.2765	0.2160	0.3801	0.3203	0.0013
17q22	304	rs7214685-rs8071417	45763 (5)	0.2687	0.1940	0.3871	0.3313	0.0005
17q22	305	rs721427-rs9652852	116730 (21)	0.2586	0.1995	0.4003	0.3313	0.0009
17q22	306	rs16969033-rs4794718	83871 (15)	0.2547	0.1995	0.4003	0.3368	0.0022
17q22	307	rs8071867-rs12949540	148635 (33)	0.2562	0.1940	0.4019	0.3423	0.0015
17q22	308	rs12601123-rs4793550	51316 (11)	0.2516	0.1885	0.4003	0.3643	0.0094
17q22	309	rs2111016-rs1024819	26322 (5)	0.2523	0.1830	0.4019	0.3588	0.0038
17q22	311	rs3744089-rs2190759	92443 (14)	0.2375	0.1885	0.4143	0.3698	0.0240
17q22	312	rs4793565-rs203257	44871 (10)	0.2368	0.1830	0.4151	0.3698	0.0180
17q22	313	rs10515149-rs4793574	81338 (16)	0.2360	0.1775	0.4213	0.3917	0.0285
17q22	314	rs9894704-rs2586083	77312 (13)	0.2079	0.1665	0.4517	0.3972	0.0158
17q22	315	rs7207440-rs16942637	6718 (3)	0.2048	0.1665	0.4533	0.4027	0.0291
17q22	316	rs2585842-rs2109248	107102 (9)	0.2048	0.1720	0.4548	0.4027	0.0369
17q22	317	rs7211774-rs2070107	165218 (18)	0.2072	0.1775	0.4556	0.4027	0.0423
17q22	318	rs13414-rs41346650	1007740 (40)	0.2095	0.1720	0.4541	0.4082	0.0372
17q22	319	rs1868916-rs10515177	723115 (40)	0.2235	0.1720	0.4439	0.4082	0.0274
17q22	320	rs9303417-rs9890799	527113 (31)	0.2150	0.1940	0.4541	0.3917	0.0334
17q22	321	rs11655927-rs9908090	494518 (30)	0.2150	0.1885	0.4517	0.3917	0.0252

Table S3 Software used in this study. A summary listing web URLs, version information and important parameter settings of software used in this study.

Program	Web URL	Version	Parameters
**SHAPEIT**	https://mathgen.stats.ox.ac.uk/genetics_software/shapeit/shapeit.html	v2.r727	NCBI build 36 release 22 was used as genetic map
**admixture_sim.py**	http://students.washington.edu/jeanm5/		See Simulation subsection of Materials and Methods
**LAMP-LD**	http://lamp.icsi.berkeley.edu/lamp/lampld/	v1.0	*win-size=20 nr-founders=25*
**RFMix**	https://sites.google.com/site/rfmixlocalancestryinference/	v1.0.2	A window size of 0.2 cM and 10 generations were used
**ADMIXTURE**	http://www.genetics.ucla.edu/software/admixture/	1.21	*K=5*
**PLINK**	http://pngu.mgh.harvard.edu/∼purcell/plink/	v1.07	*–indep-pairwise 50 10 0.1* was used for LD filtering
**Biofilter**	http://ritchielab.psu.edu/ritchielab/software/biofilter-downloads/	2.1.0	LOKI database was built on 5 Dec 2013
**R**	www.r-project.org	3.1.0	*cor.test()* was used to estimate Pearson's correlation coefficient
**ggplot2 R package**	http://cran.r-project.org/web/packages/ggplot2/index.html	2.1.0.0	
**hexbin R package**	http://cran.r-project.org/web/packages/hexbin/index.html	1.27.0	Used to create supplementary figure 5
**hierfstat R package**	http://cran.r-project.org/web/packages/hierfstat/index.html	0.04-10	The *wc()* function was used to estimate pairwise F_{ST}
**lme4 R package**	http://cran.r-project.org/web/packages/lme4/index.html	1.1-6	The *lmer()* function was used
**lmerTest R package**	http://cran.r-project.org/web/packages/lmerTest/index.html	1.1-6	Used for obtaining *lmer* p-values

Table S4 Genetic distances between the source populations of the SAC. Pairwise F_{ST} (fixation index) values between each pair of SAC source populations are summarized in this table. F_{ST} was estimated using autosomal SNPs from the source populations described in table 5.

	**YRI**	**CEU**	**GIH**	**JPT+CHB**
**SAN**	0.0918	0.185	0.173	0.216
**YRI**		0.132	0.119	0.162
**CEU**			0.034	0.108
**GIH**				0.074
